# An Innovative Thinking-Based Intelligent Information Fusion Algorithm

**DOI:** 10.1155/2013/971592

**Published:** 2013-06-17

**Authors:** Huimin Lu, Liang Hu, Gang Liu, Jin Zhou

**Affiliations:** ^1^School of Computer Science and Technology, Jilin University, Changchun 130012, China; ^2^School of Software, Changchun University of Technology, Changchun 130012, China

## Abstract

This study proposes an intelligent algorithm that can realize information fusion in reference to the relative research achievements in brain cognitive theory and innovative computation. This algorithm treats knowledge as core and information fusion as a knowledge-based innovative thinking process. Furthermore, the five key parts of this algorithm including information sense and perception, memory storage, divergent thinking, convergent thinking, and evaluation system are simulated and modeled. This algorithm fully develops innovative thinking skills of knowledge in information fusion and is a try to converse the abstract conception of brain cognitive science to specific and operable research routes and strategies. Furthermore, the influences of each parameter of this algorithm on algorithm performance are analyzed and compared with those of classical intelligent algorithms trough test. Test results suggest that the algorithm proposed in this study can obtain the optimum problem solution by less target evaluation times, improve optimization effectiveness, and achieve the effective fusion of information.

## 1. Introduction 

Information fusion [1] refers to the process in which relevant information is searched and extracted from multiple distributed heterogeneous network resources and then converted into a unified knowledge mode. It aims at constructing effective knowledge resources for solving the problems in certain field or generating new integrative knowledge object by conversing, integrating, combining, and so forth various information coming from distributed information resources. 

Common information fusion algorithms can be divided into two main categories, which are probability statistics method and artificial intelligence method. Probability statistics method includes Bayes, the transformation of Bays [2], and D-S evidence reasoning [3]. It has axiomatic basis and low computational complexity and is intuitive and easy to be understood, but it needs more prior information and its applicable condition is harsher; while in artificial intelligence method, information fusion is similarly regarded as that human brain comprehensively treats information. In this method, artificial neural network [4], support vector machine [5], and genetic algorithm (GA) [6] account for approximately 85% of the whole information fusion algorithm. And the machine learning methods, that is, swarm intelligence, artificial immune, quantum genetic algorithm, and so forth, have been applied in information fusion. This method shows fewer requirements to the prior information of object and stronger self-fitness. Moreover, the fusion of the subjective and objective information in system can be realized using this method. Most of existing intelligent algorithms are proposed based on natural evolution rule, animal collective intelligence, and life system mechanism. However, they fail to make good use of the background factors of problems and the knowledge produced in the process of solving problems. This situation limits the natural combination of intelligent algorithm and knowledge to some extent and the full play of the role of knowledge.

The researches on information fusion currently show a developing trend of further combining with the cognitive system-based human natural intelligence. The cognitive behaviors of humans, such as learning and reasoning, handle various information by widely simulating human intelligence from each link of information carrier to fusion based on knowledge [7]. Innovative thinking is the advanced stage of thinking activities. It is a thinking process of discovering new things, creating new methods, and solving new problems on the basis of personal experiences. Moreover, it is the source of wisdom [8, 9]. Through modeling and simulating innovative thinking by computer, the innovative ability on human level can be obtained and produced. By simulating human thinking mechanism, it is expected to reach a higher solving efficiency than that obtained by the natural selection mechanism-based intelligent algorithm [10].

Therefore, in references to the relevant research achievements of brain cognitive theory and creativity computation, this study puts forward an intelligent algorithm which can realize information fusion—innovative thinking-based intelligent information fusion algorithm (ITIIF algorithm) by the algorithmization to cognitive process and behavior of innovative thinking.

## 2. The Basic Framework of ITIIF Algorithm

**Figure 1:**
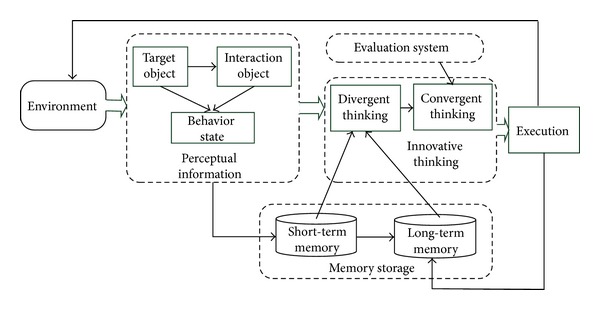
The basic framework and the relationship of the five parts of ITIIF algorithm.

ITIIF algorithm treats knowledge as core and information fusion as a knowledge-based innovative thinking process. It gives full play to the role of the knowledge-based innovative thinking skills in information fusion. This algorithm is composed by five parts, which are information sense and perception, memory storage, innovative thinking, evaluation system, and execution. Among which, innovative thinking includes two thinking skills, which are divergent thinking and convergent thinking. The basic framework and the relationship of the five parts of this algorithm are shown in Figure 1.

ITIIF algorithm can be expressed by sextuple as

(1) $$\begin{array}{c} \text{ITIIF}=\langle P,M,D,C,V,E\rangle , \end{array}$$

where *P* is the perceptual information module, *M* is memory storage module, *D* is divergent thinking module, *C* is convergent thinking module, *V* is evaluation system module, and *E* is execution module. The functions of each module are as follows.

1. Perceptual information module: it reasons and fuses the information collected from environment and maps this information into knowledge. It includes information sense and information perception.
2. Memory storage module: it organizes and manages knowledge; it includes long-term memory (LTM) and short-term memory (STM).
3. Divergent thinking module: it integrates the physical world information world obtained and information world knowledge into a structural knowledge divergent model. This process can be regarded as a self-organizing process of the knowledge in human brain. It assembles the knowledge fragments into a knowledge mode with certain structure (tree or figure).
4. Convergent thinking module: it transforms the knowledge divergent model produced by divergent thinking into decision by focus, modification, or innovation.
5. Evaluation system module: it is the assessment standard for the decision satisfaction degree. It is used to evaluate the whole or part of decision plan. In this module, value system will affect thinking modules.
6. Execution module: it applies the decision produced by thinking into environment to realize intelligent control.

## 3. The Modules of ITIIF Algorithm

### 3.1. Information Sense and Information Perception

(1) Information sense: it means that certain attributive character or behavior states of the target object collected are processed according to rule and historical data to acquire accurate values. It is represented as

(2) $$\begin{array}{c} \text{F-sense}\;\left(A,B,C,\ldots \right)=\text{S}1\left(a,b,c,\ldots \right). \end{array}$$

F-sense (*A*, *B*, *C*, …) denotes the processing of information sense, where *A*, *B*, and *C* are the sets of the multiple values of certain sense information collected and *a*, *b*, and *c* are the accurate values obtained by sense information processing.

For example, the temperature information in a room is obtained by multiple temperature sensors distributed in different positions *T* = (21°, 22°, 37°, 24°, 23°). Firstly, based on rules, there is *t* ∈ [*t*_0_ − *δ*, *t*_0_ − *δ*], where *t* is room temperature, *t*_0_ is the historical data measured, and *δ* is temperature range control parameter. Assume that *t*_0_ = 22°, *δ* = 5°, 37° will be deleted as an outlier. Then, based on averaging rule, the current room temperature is calculated as *t* = (21° + 22° + 24° + 23°)/4 = 22.5°.

(2) Information perception: it maps a plurality of sense information into knowledge cluster by reasoning and integrating. It is expressed as

(3) $$\begin{array}{c} \text{F-perception:}\;\text{S}1\left(a,b,c,\ldots \right)\to \text{KC}, \end{array}$$

where F-perception: S1(*a*, *b*, *c*, …) is the processing of cognitive information and KC refers to knowledge cluster.

**Definition 1 (knowledge cluster (KC))**

It refers to the knowledge structure connected by target object (including attribute and behavior) and related objects; it is expressed as

(4) KC={tar_o,(ro1,ro2,…,roi),(r1,r2,…,ri) ∣ roi∈O,ri∈Re},

where tar_o represents target object, ro_*i*_ is related object, O is object set, and *Re* is the set of the relationships of target object and related objects.

**Figure 2:**
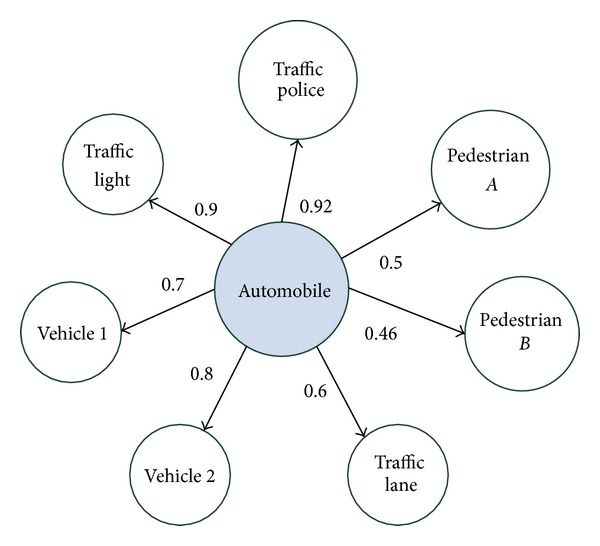
The knowledge cluster formed with an automobile as object.

For example, in a traffic intersection, the KC formed with an automobile as object is shown in Figure 2.

Thus the knowledge cluster in Figure 2 can be expressed as {automobile, (traffic police, pedestrian A, pedestrian B, traffic lane, vehicle 2, vehicle 1, traffic light), (0.92, 0.5, 0.46, 0.6, 0.8, 0.7, 0.9)} (Algorithm 1).

### 3.2. Memory Storage

Memory storage is divided into LTM and STM; it is denoted as

(5) $$\begin{array}{c} \text{M}=\langle \text{LTM},\text{STM}\rangle \end{array}$$

STM is used to store the knowledge cluster constructed by information perception as well as thinking process data. LTM is used to store the knowledge unit produced by innovative thinking. It can carry out the automatic evolution of knowledge.

#### 3.2.1. STM

STM is a temporary memory space opened in thinking process for the development of thinking activities. STM can be expressed as

(6) $$\begin{array}{c} \text{STM}=\left\{\text{k}{\text{c}}_{i}\;|\;i=1,2,\ldots ,n\right\}, \end{array}$$

where kc_*i*_ represents an independent knowledge cluster, kc_*i*_ = 〈*D*_kc_*i*__, *t*_kc_*i*__〉, *D*_kc_*i*__ denotes the stimulation intensity of KC, which suggests the influencing degree of KC on target environment. Bigger *D*_kc_*i*__ explains that KC cannot be easily forgotten; *t*_kc_*i*__ is the existing time of KC in STM. The greater the *t*_kc_*i*__ is, the more easily the knowledge cluster can be forgotten.

#### 3.2.2. LTM

LTM is the container of knowledge units. The knowledge units obtained by innovative thinking are stored in LTM.

**Definition 2 (knowledge unit)**

It refers to the knowledge structure connected by subobjects in sequence to achieve the expected state of system. It can be expressed as

(7) KU={(sgi,sgj,rij) ∣ sgi,sgj∈Ω,rij∈SGRe},

where *Ω* is subtarget space, *SG*_*Re*_ denotes the set of the interaffecting relationship of *sg*_*i*_ and *sg*_*j*_ to realize the expected effect of system.

LTM is expressed as

(8) $$\begin{array}{c} \text{LTM}=\left\{\text{k}{\text{u}}_{i}\;|\;i=1,2,\ldots ,n\right\}. \end{array}$$

ku_*i*_ is an independent knowledge unit, ku_*i*_ = 〈*F*_ku_*i*__, *t*_ku_*i*__〉, *F*_ku_*i*__ is the fitness of knowledge unit. Larger *F*_ku_*i*__ indicates a stronger activeness of knowledge unit, and this knowledge unit can more easily be extracted by thinking module. *t*_ku_*i*__ refers to the time that knowledge unit is stored in LTM. Larger *t*_ku_*i*__ suggests that the knowledge unit will be more easily forgotten.

#### 3.2.3. Knowledge Evolution

The knowledge units in LTM continuously evolve under the effect of innovative thinking activities. This evolution contains quality development and quantity growth. The quantity growth of knowledge represents the increase of total knowledge quantity in LTM after a certain period. The quality development of knowledge refers to the improvement of the depth and truth degree of the recently emerged knowledge units produced by innovative thinking comparing with that in certain past historical period. By quality development of knowledge, the knowledge units in LTM can be updated and continuously evolve.

The specific steps of knowledge evolution are as follows.

1. Initialize knowledge evolution scale *Pk*.
2. *list*(ku_1_, ku_2_, ku_3_,…, ku_*n*_) //*list*() sorts the knowledge units in LTM from high to low by fitness, ku_*n*−1_.*fitness*() > ku_*n*_.*fitness*(), where *fitness*(ku_*j*_) = *w*_1_ *p*_*j*1_ + *w*_2_ *p*_*j*2_ + ⋯+*w*_*n*_ *p*_*jn*_, *p*_*j*1_, *p*_*j*2_,…, *p*_*jn*_ is the evaluation value of the 1, 2,…, *n* subtarget in the *j* knowledge unit, and *w*_*n*_ is weight.
3. *select*(ku_1_, ku_2_, ku_3_,…, ku_*pk*_) //*select*() select the first *Pk* knowledge units into evolution pool.
4. The knowledge units in evolution pool are stochastically paired 〈ku_*i*_, ku_*j*_〉, and >*j*,  *i* = 1,2,…, *pk*, *j* = 1,2,…, *pk*.
5. By the recombination and local mutation to the attribute gene corresponded to the chromosome of knowledge unit, new knowledge unit is generated {ku_new1_, ku_new2_,…, ku_new*n*_}.
6. Test the effectiveness of new knowledge units: if ku_new*n*_ · *effectiveness*() > Th, this new knowledge unit is stored in LTM, where Th is threshold. *effectiveness*(ku_new*n*_) = *w*_1_ *A*(ku_new*n*_) + *w*_2_ *B*(ku_new*n*_) + *w*_3_ *C*(ku_new*n*_), where *A*(ku_new*n*_) is correctness of knowledge unit, *B*(ku_new*n*_) is the coverage degree of knowledge unit, and *C*(ku_new*n*_) is the reliability of knowledge unit.

*A*(ku_new*n*_) measures the degree of knowledge unit ku_new*n*_ realizing the expected target of system, *A*(ku_new*n*_) = *est*(). *est*() is target estimation function. Its estimation accuracy is related to the precision of knowledge and estimation method.

*B*(ku_new*n*_) represents the ratio of the number of sub-target contained in ku_new*n*_ and the number of sub-target contained in realizing the expected target. *B*(ku_new*n*_) = ∑_*i*=1_^|*SG*_ku_new*n*__|^(*sg*_*i*_ ∈ *SG*_ku_new*n*__∩*SG*_*Ω*_)/|*SG*_*Ω*_|, where *SG*_ku_new*n*__ is the sub-target contained in knowledge unit ku_new*n*_, |*SG*_ku_new*n*__| is the cardinal number of *SG*_ku_new*n*__, and *SG*_*Ω*_ denotes the sub-target contained in realizing the expected target of system in theory.

*C*(ku_new*n*_) is the correctness degree of knowledge unit, *C*(ku_new*n*_) = (1 + ((*T*_*Ω*_ − 1)/*T*_LTM_))/*T*_*Ω*_; in this formula, *T*_*Ω*_ indicates the number of knowledge unit in realizing the expected target of system in theory; *T*_LTM_ is the number of knowledge unit in realizing expected target of system contained in LTM.

### 3.3. Divergent Thinking

Divergent thinking is a process of assembling the sub-targets in different knowledge units to produce new target path according to the characteristics of problem and the process required in assembling knowledge units [11]. It extracts and assembles certain knowledge units in memory storage to achieve the reflection from LTM to knowledge tree. It is represented as

(9) $$\begin{array}{c} \text{divergent: LTM}\to \text{KST}, \end{array}$$

where KST refers to the knowledge structure tree assembled; divergent composes two processes, which are memory extraction and knowledge assembling.

#### 3.3.1. Memory Extraction

The extraction of knowledge units is controlled by the fitness *F*_ku_*i*__ of the knowledge units and existing time *t*_ku_*i*__. And the memory extraction model is established as

(10) $$\begin{array}{c} GK\left(\text{k}{\text{u}}_{i}\right)=F_{\text{k}{\text{u}}_{i}}{\text{e}}^{-\lambda t_{\text{k}{\text{u}}_{i}}}, \end{array}$$

where *GK*(ku_*i*_) is the extraction intensity of the *i* knowledge unit. Larger *GK*(ku_*i*_) suggests that knowledge unit can more possibly be extracted at divergent thinking stage. Divergent thinking will extract *C*_LTM_ knowledge units with larger *GK*(ku_*i*_) value from LTM. *C*_LTM_ is the extraction capacity of knowledge unit; it determines the number of the maximum knowledge units that can be extracted from LTM. 

#### 3.3.2. Knowledge Assembling

Knowledge assembling combines the knowledge units extracted by memory into knowledge structure tree (KST). Several definitions that are related to KST are shown in the following.

**Definition 3 (knowledge structure tree)**

It refers to the data structure produced by combining the knowledge units in certain order according to the characteristics of problem. It is presented as

(11) $$\begin{array}{c} \text{KST}=\langle g,SG,\text{DB},P\rangle , \end{array}$$

where *g* is target node; it reflects the purposiveness of divergent thinking. There is only one target node in KST; *SG* is sub-target node set; it refers to all subtargets that are related to problem solution; DB is directed border set; *P* is the relationship parameter maintained by each node.

**Definition 4 (knowledge path)**

If there is a node sequence {node_*m*_, node_1_, node_2_,…, node_*k*_, node_*n*_} in KST and correlations *parentOf* (node_*m*_, node_1_), *parentOf* (node_1_, node_2_), and *parentOf* (node_*k*_, node_*n*_) belong to KST, there is a knowledge path *P*(node_*m*_, node_*n*_) between node_*m*_ and node_*n*_.

*pa* *re* *nt* *Of* (node_*i*_, node_*j*_) refers to that node_*i*_ is the parent node of node_*j*_.

**Definition 5 (knowledge radius)**

If there is a knowledge path *P*(node_*m*_, node_*n*_) between node_*m*_ and node_*n*_ in KST, the node conversion times on this path are called the distance between node_*m*_ and node_*n*_. And the shortest distance is the knowledge radius of node_*m*_ and node_*n*_.

**Figure 3:**
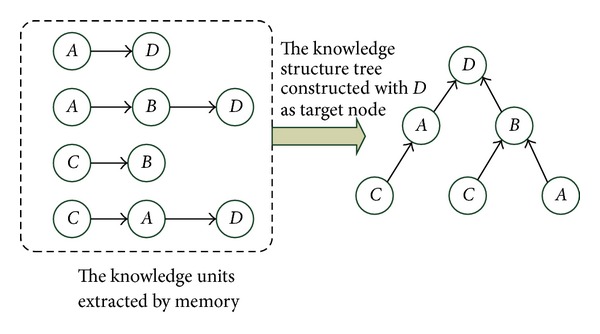
The assembling process of knowledge structure tree.

Figure 3 presents the assembling process from knowledge unit to KST.

Each knowledge path from source node to target node represents one problem solving plan. Source node is the sub-target node containing no subnodes. Divergent thinking is, namely, a process of listing all problem solving plans according to existing knowledge level and cognitive conditions. Every knowledge path can be regarded as a special kind of knowledge unit. But there is no one-to-one corresponding relationship between knowledge path and knowledge unit. All knowledge units can find their corresponding knowledge paths in KST, but not all knowledge paths in KST can find their corresponding knowledge units. This phenomenon suggests that divergent thinking has combining and innovating capacity. For example, the knowledge unit extracted by the knowledge path *C* → *B* → *D* in KST from memory does not exist, it is generated by the combination of knowledge units *C* → *B* and *A* → *B* → *D*. That is, the combination of *C* → *B* and *A* → *B* → *D* creates a problem solution plan *C* → *B* → *D*.

Knowledge assembling constructs KST based on the method of knowledge radius and with knowledge target as center. The specific KST construction algorithm is as follows (see Algorithm 2).

### 3.4. Convergent Thinking

Multiple problem solving plans have been produced through assembling and innovating knowledge units by divergent thinking, while there is a focus process when human's thinking proceeds to certain stage. After focusing, usually several limited objects are retained. This is the process of convergent thinking. Convergent thinking refers to the process that the divergent knowledge model formed by divergent thinking is transformed into new problem solving plans by focusing, modifying, and innovating. It realizes the reflection from KST to the optimum problem solving plan. It is indicated as

(12) $$\begin{array}{c} \text{convergent: KST}\to \text{CS}. \end{array}$$

#### 3.4.1. Convergent Operation Focus()

Focus() can select several paths with higher values form multiple knowledge paths of KST. Its convergent rule is

(13) $$\begin{array}{c} F=\langle f\left(\text{k}{\text{p}}_{i}\right),n_{\text{con}}\rangle , \end{array}$$

where *f*(kp_*i*_) is focus function, kp_*i*_ ∈ KST represents all knowledge paths included in KST, *n*_con_ is convergent dimension, namely, the number of the knowledge paths focused.

Focus function *f*(kp_*i*_) : KST → [0,1] maps all the knowledge paths in KST into *p* ∈ [0,1]. *p* reflects the focused probability of each knowledge path. The knowledge path with larger *p* suggests that the probability of this knowledge path being in focus is higher. The knowledge path with higher values in KST poses higher convergent probability. Thus *f*(kp_*i*_) = *V*_kp_*i*__(KST); *V*_kp_*i*__(KST) is the values of the *i* knowledge path in KST.

#### 3.4.2. Modified Operation: Modified()

When the problem solving value achieved by the focused knowledge path shows little differences with expected target value, modified() is operated. It is represented as

(14) $$\begin{array}{c} \text{modified}\left(sg_{i},G,E,R\right), \end{array}$$

where *sg*_*i*_ is the sub-target modified; *G* is the expected target achieved by implementing modified operation; *E* is the environment of modified operation, namely, the affecting relationship of *G* and the integrity degree of the sub-targets except *sg*_*i*_ in focused knowledge path. *R* is resource restriction. It refers to the usable resources in the premise of ensuring the resource demand of other sub-targets.

#### 3.4.3. Innovation Operation: Innovate()

When the problem solving value achieved by the focused knowledge path shows a larger difference with expected target value, innovate() is conducted. It is expressed as

(15) $$\begin{array}{c} sg_{\text{new}}=\text{innovate}\left(\text{FK}{\text{P}}_{sg_{i}}\right), \end{array}$$

where *sg*_new_ ∈ *Ω* is the new target after innovation. FKP_*sg*_*i*__ is the *i* sub-target on focused knowledge path.

Aiming at different problems and different problem solving stages, human thinking will choose different innovation styles (modified operation or innovation operation) according to environment changes:

(16) $$\begin{array}{c} \text{MI}\left(\text{kp},\text{cre}\_\text{style},v_{e},\alpha ,\beta \right), \end{array}$$

where kp is the knowledge path produced by focus operation, cre_style ∈ {0,1} is creative style. cre_style = 1 refers to dynamic creative style parameter; cre_style = 0 is static creative style parameter; *v*_*e*_ is target expected value; *α* ∈ [0,1] is modified operation coefficient; *β* = 1 − *α* is innovation operation coefficient. If cre_style = 1, the style is dynamic creative style; its parameter is defined as

(17) $$\begin{array}{c} \alpha^{*}=1-\frac{v_{e}-v}{v_{e}},\;\;\beta^{*}=1-\alpha^{*}, \end{array}$$

where *v* refers to the value that can be achieved by current knowledge path. It can be known that, with the increase of *v*, the probability of implementing modified innovation is gradually enhanced, while the probability of implementing innovative innovation is gradually weakened.

### 3.5. Evaluation System

Evaluation system aims at evaluating the satisfaction degree to problem solving plan, which is, evaluating the values of the knowledge paths contained in KST. The satisfaction degree of the *i* knowledge path is denoted as

(18) $$\begin{array}{c} \text{SAT}\left(\text{KS}{\text{T}}_{\text{k}{\text{p}}_{i}}\right)=1-\frac{V_{ge}-V\left(\text{KS}{\text{T}}_{\text{k}{\text{p}}_{i}}\right)}{V_{ge}}, \end{array}$$

where *V*_*ge*_ is expected values of target; it is decided by decision makers; *V*(KST_kp_*i*__) is the values of the *i* knowledge path in KST. The specific calculation formula is as follows:

(19) V(KSTkpi)=w1V(kpi(sg1))+w2V(kpi(sg2))+⋯+wjV(kpi(sgj)).

*w*_*j*_ is weight; *V*(kp_*i*_(*sg*_*j*_)) is the contribution value provided by the *j* sub-target in the *i* knowledge path in achieving the total target; *V*(kp_*i*_(*sg*_*j*_) = *f*(*t*_*sg*_*j*__, *l*_*sg*_*j*__, *n*_*sg*_*j*__), *f*(*t*_*sg*_*j*__, *l*_*sg*_*j*__, *n*_*sg*_*j*__) are the evaluation functions related to the memory time, the layer number in KST, and the subnodes number of sub-target *j*. *V*(kp_*i*_(*sg*_*j*_)) is closely connected with knowledge expression form. Therefore, the calculation mode of *f*(*t*_*sg*_*j*__, *l*_*sg*_*j*__, *n*_*sg*_*j*__) should be instantiated when facing specific problem.

## 4. ITIIF Algorithm Realization

According to the analysis on each module of ITIIF algorithm, the specific realization of ITIIF algorithm is explained in Algorithm 3.

## 5. Experiment and Analysis

### 5.1. Experiment and Analysis on the Parameters of ITIIF Algorithm

The experimental data employ the data in “The Famous Knowledge Base for OpenCyc”. By sorting, test data set is formed. It includes 37,310 knowledge units and 6, 531 correlations between knowledge units. This test aims at analyzing the parameters (*C*_LTM_, *n*_con_, cre_style) in ITIIF algorithm using knowledge navigation path optimization problem. First, we define two indexes—thinking set degree and information quantity.

**Definition (thinking set degree)**

It refers to the aggregation degree of the solutions obtained by convergent thinking. It is expressed as

(20) $$\begin{array}{c} \text{SE}\left(t\right)=\sum_{i}\frac{k_{i}\left(t\right)}{t}\ln\frac{k_{i}\left(t\right)}{t}, \end{array}$$

where *t* is evolution generation number, SE(*t*) is the thinking set degree of the *t* generation. *k*_*i*_(*t*) is the total focused times of the *i* solution that has been focused up to the *t* generation.

IQ(*t*) refers to the information quantity obtained after evolution of *t* generations. It is expressed as

(21) $$\begin{array}{c} \text{IQ}\left(t\right)=\frac{n_{f}\left(t\right)}{2n^{2}}, \end{array}$$

where *n*_*f*_(*t*) refers to the different knowledge unit number in current LTM. *n* is the knowledge unit number in corpus.

**Figure 4:**
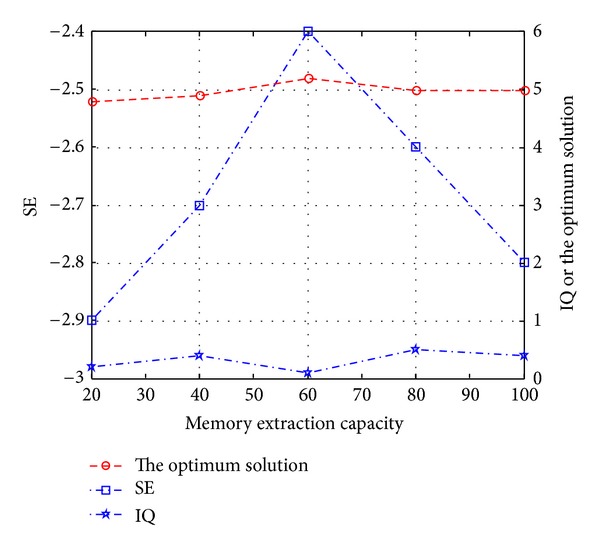
The relationships of *C*_LTM_ with thinking set degree, information quantity, and the optimum solution.

**Figure 5:**
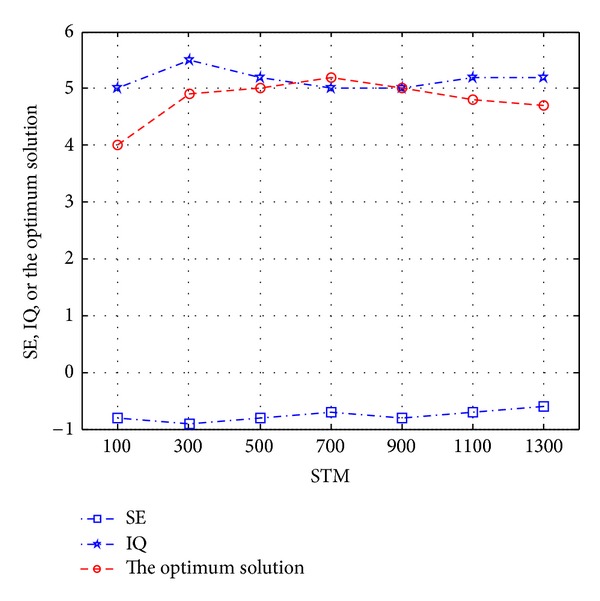
The relationships of STM with thinking set degree, information quantity, and the optimum solution.

**Figure 6:**
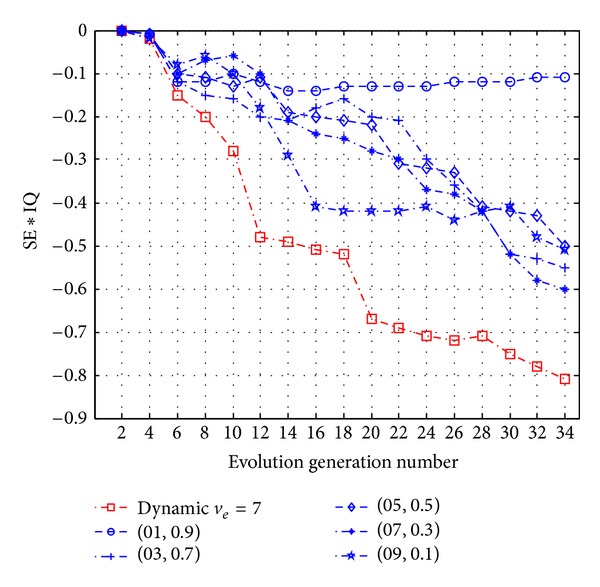
The relationship between creative style with SE(*t*)∗IQ(*t*).

The influences of parameter *C*_LTM_, *n*_con_, cre_style on the performance of ITIIF algorithm are shown in Figures 4, 5, and 6, respectively.

As shown in Figure 4, *C*_LTM_ shows great influences on the optimization effect of navigation path. In view of the whole process, problem solving effect experiences a process that first increases then decreases with the increase of *C*_LTM_; on one hand, with the increase of *C*_LTM_, thinking set degree SE(*t*) first increases and then decreases. SE(*t*) increase indicates that convergent thinking is easy to “focus” on certain solution with a relatively small number; on the other hand, with the increase of *C*_LTM_, information quantity IQ(*t*) firstly increases then decreases and increases again. Information quantity increase suggests that the information required in solving the problem is perceived more completely and deeply. Meanwhile, there is a law that the target value of optimum solution is proportional to SE(*t*)∗IQ(*t*).

In Figure 5, with the increase of convergence dimension *n*_con_, optimization effect of problem solving first reaches a certain value and then decreases. This phenomenon suggests that, with the increase of information quantity, the divergent degree of thinking will increase. But when the divergent degree is too high, problem solving effect will be influenced.

As shown in Figure 6, as for dynamic creative style cre_style = 1, better optimization effect can be obtained when the *v*_*e*_ selected is close to the optimum value. When the *v*_*e*_ selected is deviated from the optimum value, the performance of ITIIF algorithm reduces greatly; while as for the static creative style cre_style = 0, the optimizing capacity of ITIIF algorithm is continuously improved with the increase of *β*. In general, the SE(*t*)∗IQ(*t*) value obtained when dynamic creative style parameter is applied is lower than that when static creative style parameter is applied. So it can be concluded that if the probable range of the optimum value of target can be predicted, it is proper to select dynamic creative style; if the range of target value cannot be confirmed, static creative style is more proper. At the same time, the global optimization capacity of ITIIF algorithm can be improved by increasing *β* value. Commonly, *β* is set in 0.6 to 0.9.

### 5.2. Comparison Test

#### 5.2.1. Comparison of Optimization Results

To better display the performance of ITIIF algorithm, this paper adopts five testing functions to develop simulation tests. Moreover, the simulation results of ITIIF algorithm are compared with those of the currently used binary particle swarm and binary differential evolution algorithm.  Table 1 lists the test functions.

To provide comparability, the evaluation indexes in this test employ mean best fitness (MBF) and standard deviation (SD). MBF reflects the accuracy that algorithm can achieve when iteration times are given. SD reflects the stability and robustness of algorithm.  Table 2 shows the solution results of the testing functions by ITIIF algorithm, binary particle swarm algorithm, and binary differential evolution algorithm. The values of MBF and SD are obtained by independently running each algorithm for 20 times, respectively. 

It can be seen from Table 2 that the ITIIF algorithm proposed in this study shows better optimization performances to the functions, regardless of unimodal function or multimodal function. Moreover, it achieves more ideal optimization effects in terms of solution accuracy and stability. This result shows that ITIIF algorithm has certain advantages in function optimization.

#### 5.2.2. Performance Comparison

The ITIIF algorithm proposed in this study is compared with the commonly used genetic algorithms (GA), estimate distribution algorithms (EDAs), and ant colony optimization (ACO) in three aspects, which are average distance, average time, and average assessment value. The parameters settings of each algorithm are shown in Table 3.

**Figure 7:**
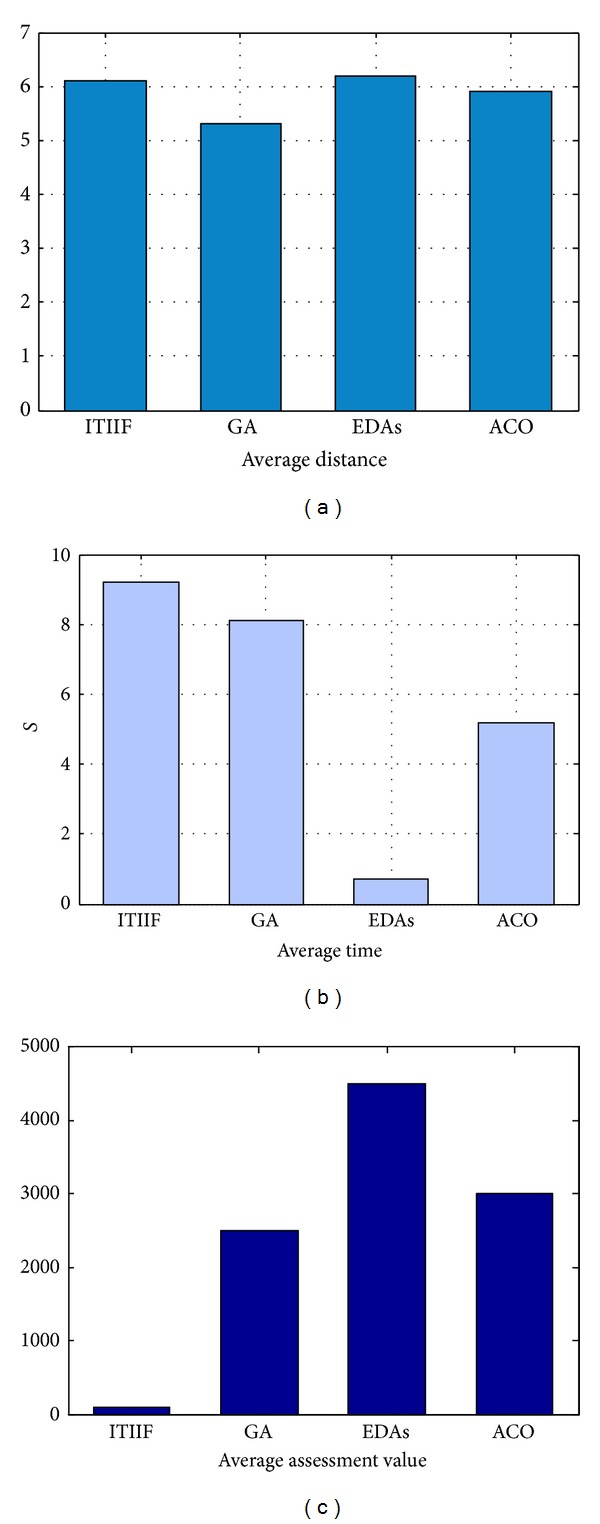
The performance comparison of ITIIF, GA, EDAs, and ACO.

The comparison results are presented in Figure 7. It can be obtained that ITIIF and EDAs show better optimization effects, while GA present the poorest optimization effect. The average time of ITIIF is longer than that of the other three algorithms. This is because the evolution of ITIIF algorithm requires learning and innovative thinking. And the longer average time of ITIIF algorithm is just the result of fully used knowledge. With the accumulation of knowledge, the time consumed in solving problem will be decreasing, just as human can improve the problem solving efficiency through continuous accumulations of knowledge and experience by learning. The assessment value of ITIIF is only 4.5% to 8% of other algorithms. This result means that the time required in target evaluation is greatly reduced.

According to above experimental results, it can be concluded that ITIIF algorithm shows better efficiency than some of current intelligent algorithms. This is because ITIIF algorithm replaces evaluation operation with creative thinking process. Thus ITIIF algorithm can make better use of the knowledge generated by target evaluation to further improve efficiency. In addition, the evolution process of ITIIF algorithm can be explained. And ITIIF algorithm directly simulates the cognitive behavior of human and is consistent with the cognitive habits of human. This is what not possessed by other intelligent algorithms.

## 6. Conclusions

In references to the research achievements of modern brain science, neurophysiology, and cognitive theories, this study proposes a knowledge-based and value evaluation-oriented information fusion algorithm starting from the viewpoints of biological control theory and macro control system theory. Besides, this algorithm integrates the skills of convergent thinking and divergent thinking and applies the structure analysis methodology of macro system control theory into the simulation and modeling of innovative thinking. The test results show that this algorithm can reduce the number of target evaluation times, improve optimization efficiency, and realize the effective fusion of information. The following work is to strengthen strict theoretical derivation, incorporate innovative thinking into the frame of this algorithm, extensively integrate this algorithm with various traditional evolutionary algorithms, and widen the application area.

## Figures and Tables

**Algorithm 1:**
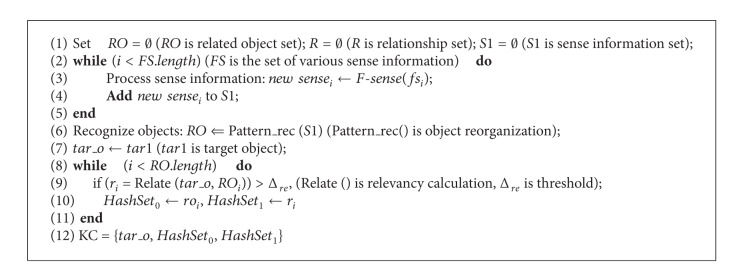
KC_construction algorithm.

**Algorithm 2:**
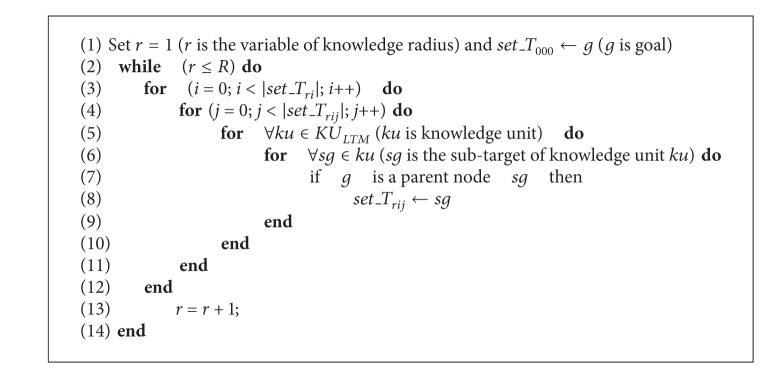
KST_construction algorithm.

**Algorithm 3:**
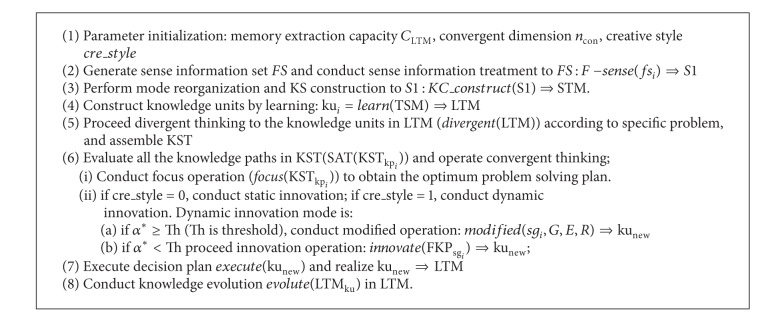
ITIIF algorithm.

**Table 1:** The test functions.

Test functions	Domain	Characteristics
*f*_1_(*x*) = ∑_*i*=1_^*n*^ *x*_*i*_^2^	$\begin{array}{c} x_{i}\in [-5.12,5.12],\\ i=1,2,\ldots ,n \end{array}$	Unimodal function, the optimum value locates on point 0

*f*_2_(*x*) = 100(*x*_1_^2^ − *x*_2_)^2^ + (1 − *x*_1_)^2^	$\begin{array}{c} x_{i}\in [-2.048,2.048],\\ i=1,2,\ldots ,n \end{array}$	Unimodal function is a nonconvex and pathological function. The global optimum value is located in a smooth, long, and narrow parabolic canyon

$f_{3}(x)=0.5+(\sin^{2}\sqrt{x_{1}^{2}+x_{2}^{2}}-0.5)/{\left[1.0+0.001(x_{1}^{2}+x_{2}^{2})\right]}^{2}$	$\begin{array}{c} x_{i}\in [-100,100],\\ i=1,2,\ldots ,n \end{array}$	Multimodal function. It shows a ring of local minimum values on the uplift in a range that is about 3.14 away from the global minimum value. It has strong oscillation properties. And its global minimum value is surrounded by local minimum values.

$f_{4}(x)=(1/4000)\sum_{i=1}^{n}{\left(x_{i}-100\right)}^{2}-\prod_{i=1}^{n}\cos((x_{i}-100)/\sqrt{i})+1$	$\begin{array}{c} x_{i}\in [-100,100],\\ i=1,2,\ldots ,n \end{array}$	Multimodal function, the global minimum value 0 is obtained on *x*_*i*_ = 0

*f*_5_(*x*) = ∑_*i*=1_^*n*^(*x*_*i*_^2^ − 10cos⁡(2*πx*_*i*_) + 10)	$\begin{array}{c} x_{i}\in [-5.12,5.12],\\ i=1,2,\ldots ,n \end{array}$	Ultra multimodal function, it contains about *n* ^11^ local minimum values

**Table 2:** The solution results of the testing functions by ITIIF algorithm, binary particle swarm algorithm, and binary differential evolution algorithm.

Function	Binary particle swarm algorithm		Binary differential evolution algorithm		ITIIF algorithm proposed in this study	
	MBF	SD	MBF	SD	MBF	SD
*f*_1_(*x*)	0.000006	0.000007	0.0	0.0	0.0	0.0
*f*_2_(*x*)	0.000256	0.000275	0.0	0.0	0.0	0.0
*f*_3_(*x*)	0.000001	0.000003	0.0	0.0	0.0	0.0
*f*_4_(*x*)	37.033964	4.886305	0.561765	0.160205	0.51602917	0.2710531
*f*_5_(*x*)	277.696409	10.621365	58.452170	10.675424	0.001025	0.000765

**Table 3:** The parameters settings of each algorithm.

Algorithm	Parameter setting
ITIIF	*C*_LTM_ = 50, *C*_STM_ = 10000, *n*_con_ = 1, cre_style = 1

GA	Populations = 80, iterations number = 50, crossover probability = 0.9, mutation probability = 0.1

EDAs	Populations = 300, iterations number = 50, selective probability = 0.2

ACO	Information heuristic factor = 0.8, expected heuristic factor = 0.6, pheromone evaporation rate = 0.5

## References

[1] Ruser H, Leon FP (2007). Information fusion-an overview. *Technisches Messen*.

[2] Messaoudi S, Messaoudi K, Dagtas S (2010). Bayesian data fusion for smart environments with heterogenous sensors. *Journal of Computing Sciences in Colleges*.

[3] Yang J, Huang HZ, Miao Q, Sun R (2011). A novel information fusion method based on Dempster-Shafer evidence theory for conflict resolution. *Intelligent Data Analysis*.

[4] Hong-bin Z Multi-sensor information fusion method based on the neural network algorithm.

[5] Wang Y, Zhang C, Luo J Study on information fusion algorithm and application based on improved SVM.

[6] Wanga Z, Leung KS, Wang J (1999). A genetic algorithm for determining nonadditive set functions in information fusion. *Fuzzy Sets and Systems*.

[7] Barsalou LW (2010). Introduction to 30th anniversary perspectives on cognitive science: past, present, and future. *Topics in Cognitive Science*.

[8] Wen G, Ding Y, Zheng Q (2003). Overview on computational creativity. *Pattern Recognition and Artificial Intelligence*.

[9] Boden MA (2004). *The Creative Mind: Myths and Mechanismsedition*.

[10] Bonissone PP, Subbu R, Eklund N, Kiehl TR (2006). Evolutionary algorithms + domain knowledge = real-world evolutionary computation. *IEEE Transactions on Evolutionary Computation*.

[11] Gabora L Cognitive mechanisms underlying the creative process.

